# Supplementary material for: The Natural Fermentation of Greek Tsounati Olives: Microbiome Analysis
Source: Foods. 2025 Jul 22;14(15):2568. doi: 10.3390/foods14152568 (PMC12345721; doi:10.3390/foods14152568)
Supplement: Supplementary file 1 [file foods-14-02568-s001.zip › Figure S1.pdf]

S1A

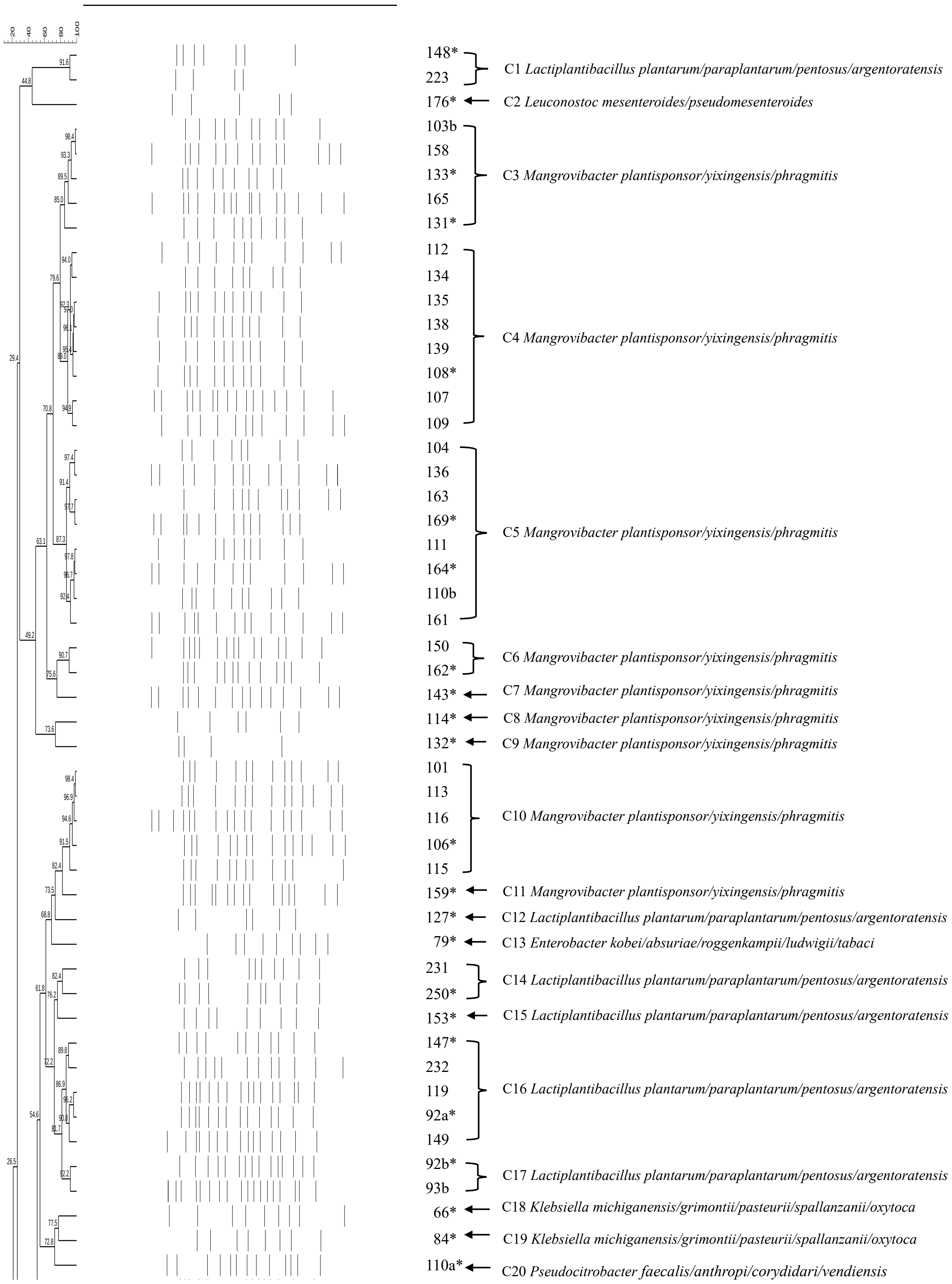
148*
223
C1 *Lactiplantibacillus plantarum/paraplantarum/pentosus/argentoratensis*
176*
C2 *Leuconostoc mesenteroides/pseudomesenteroides*
103b
158
133*
165
131*
C3 *Mangrovibacter plantisponsor/yixingensis/phragmitis*
112
134
135
138
139
108*
107
109
C4 *Mangrovibacter plantisponsor/yixingensis/phragmitis*
104
136
163
169*
111
164*
110b
161
C5 *Mangrovibacter plantisponsor/yixingensis/phragmitis*
150
162*
C6 *Mangrovibacter plantisponsor/yixingensis/phragmitis*
143*
C7 *Mangrovibacter plantisponsor/yixingensis/phragmitis*
114*
C8 *Mangrovibacter plantisponsor/yixingensis/phragmitis*
132*
C9 *Mangrovibacter plantisponsor/yixingensis/phragmitis*
101
113
116
106*
115
C10 *Mangrovibacter plantisponsor/yixingensis/phragmitis*
159*
C11 *Mangrovibacter plantisponsor/yixingensis/phragmitis*
127*
C12 *Lactiplantibacillus plantarum/paraplantarum/pentosus/argentoratensis*
79*
C13 *Enterobacter kobei/absuriae/roggenkampii/ludwigii/tabaci*
231
250*
C14 *Lactiplantibacillus plantarum/paraplantarum/pentosus/argentoratensis*
153*
C15 *Lactiplantibacillus plantarum/paraplantarum/pentosus/argentoratensis*
147*
232
119
92a*
149
C16 *Lactiplantibacillus plantarum/paraplantarum/pentosus/argentoratensis*
92b*
93b
C17 *Lactiplantibacillus plantarum/paraplantarum/pentosus/argentoratensis*
66*
C18 *Klebsiella michiganensis/grimontii/pasteurii/spallanzanii/oxytoca*
84*
C19 *Klebsiella michiganensis/grimontii/pasteurii/spallanzanii/oxytoca*
110a*
C20 *Pseudocitrobacter faecalis/anthropi/corydidari/vendiensis*

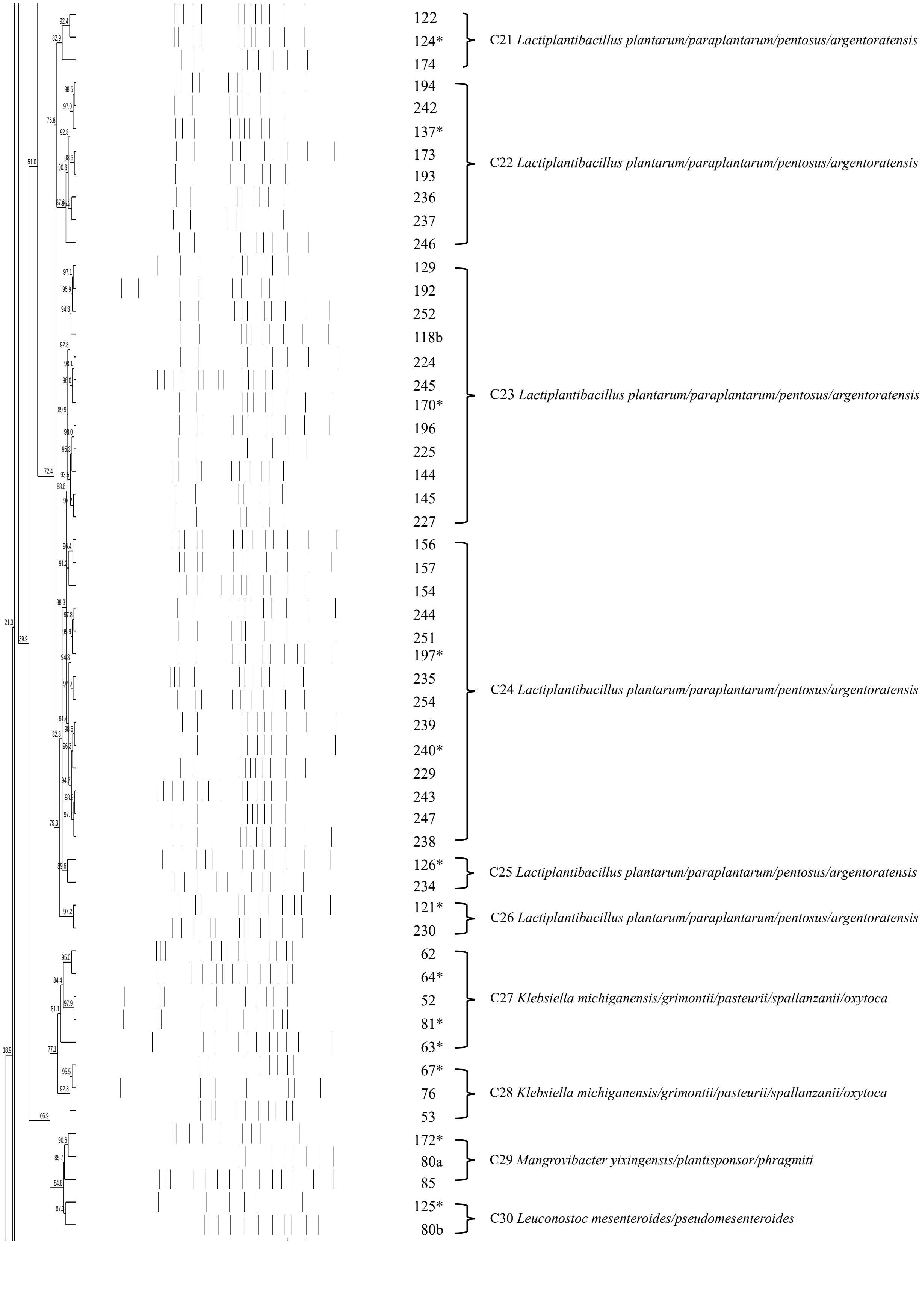
122
124*
174
C21 Lactiplantibacillus plantarum/paraplantarum/pentosus/argentoratensis
194
242
137*
173
193
236
237
246
C22 Lactiplantibacillus plantarum/paraplantarum/pentosus/argentoratensis
129
192
252
118b
224
245
170*
196
225
144
145
227
C23 Lactiplantibacillus plantarum/paraplantarum/pentosus/argentoratensis
156
157
154
244
251
197*
235
254
239
240*
229
243
247
238
C24 Lactiplantibacillus plantarum/paraplantarum/pentosus/argentoratensis
126*
234
C25 Lactiplantibacillus plantarum/paraplantarum/pentosus/argentoratensis
121*
230
C26 Lactiplantibacillus plantarum/paraplantarum/pentosus/argentoratensis
62
64*
52
81*
63*
C27 Klebsiella michiganensis/grimontii/pasteurii/spallanzanii/oxytoca
67*
76
53
C28 Klebsiella michiganensis/grimontii/pasteurii/spallanzanii/oxytoca
172*
80a
85
C29 Mangrovibacter yixingensis/plantisponsor/phragmiti
125*
80b
C30 Leuconostoc mesenteroides/pseudomesenteroides

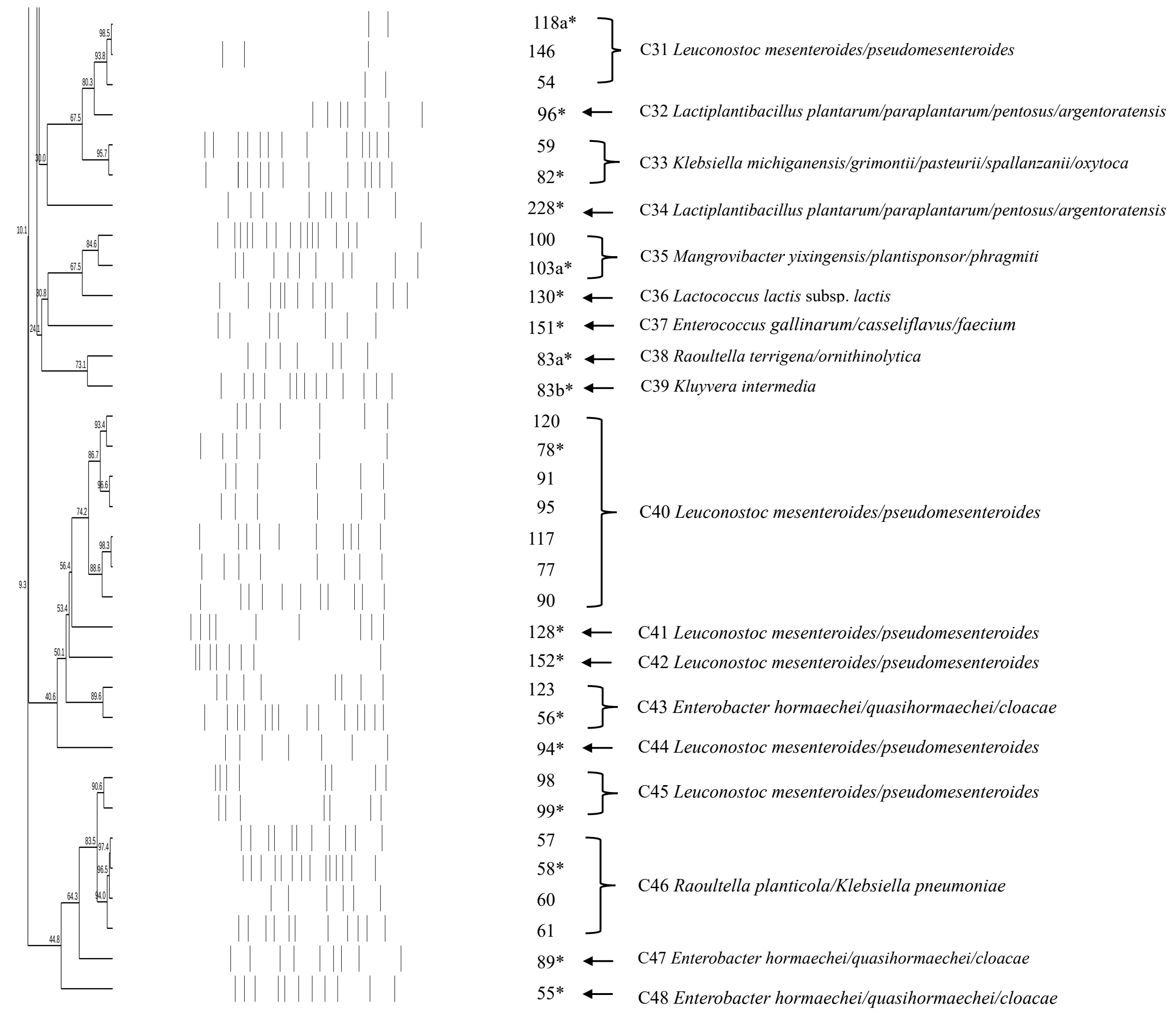

118a*
146
54
C31 Leuconostoc mesenteroides/pseudomesenteroides
96*
C32 Lactiplantibacillus plantarum/paraplantarum/pentosus/argentoratensis
59
82*
C33 Klebsiella michiganensis/grimontii/pasteurii/spallanzanii/oxytoca
228*
C34 Lactiplantibacillus plantarum/paraplantarum/pentosus/argentoratensis
100
103a*
C35 Mangrovibacter yixingensis/plantisponsor/phragmiti
130*
C36 Lactococcus lactis subsp. lactis
151*
C37 Enterococcus gallinarum/casseliflavus/faecium
83a*
C38 Raoultella terrigena/ornithinolytica
83b*
C39 Kluyvera intermedia
120
78*
91
95
117
77
90
C40 Leuconostoc mesenteroides/pseudomesenteroides
128*
C41 Leuconostoc mesenteroides/pseudomesenteroides
152*
C42 Leuconostoc mesenteroides/pseudomesenteroides
123
56*
C43 Enterobacter hormaechei/quasihormaechei/cloacae
94*
C44 Leuconostoc mesenteroides/pseudomesenteroides
98
99*
C45 Leuconostoc mesenteroides/pseudomesenteroides
57
58*
60
61
C46 Raoultella planticola/Klebsiella pneumoniae
89*
C47 Enterobacter hormaechei/quasihormaechei/cloacae
55*
C48 Enterobacter hormaechei/quasihormaechei/cloacae

S1B

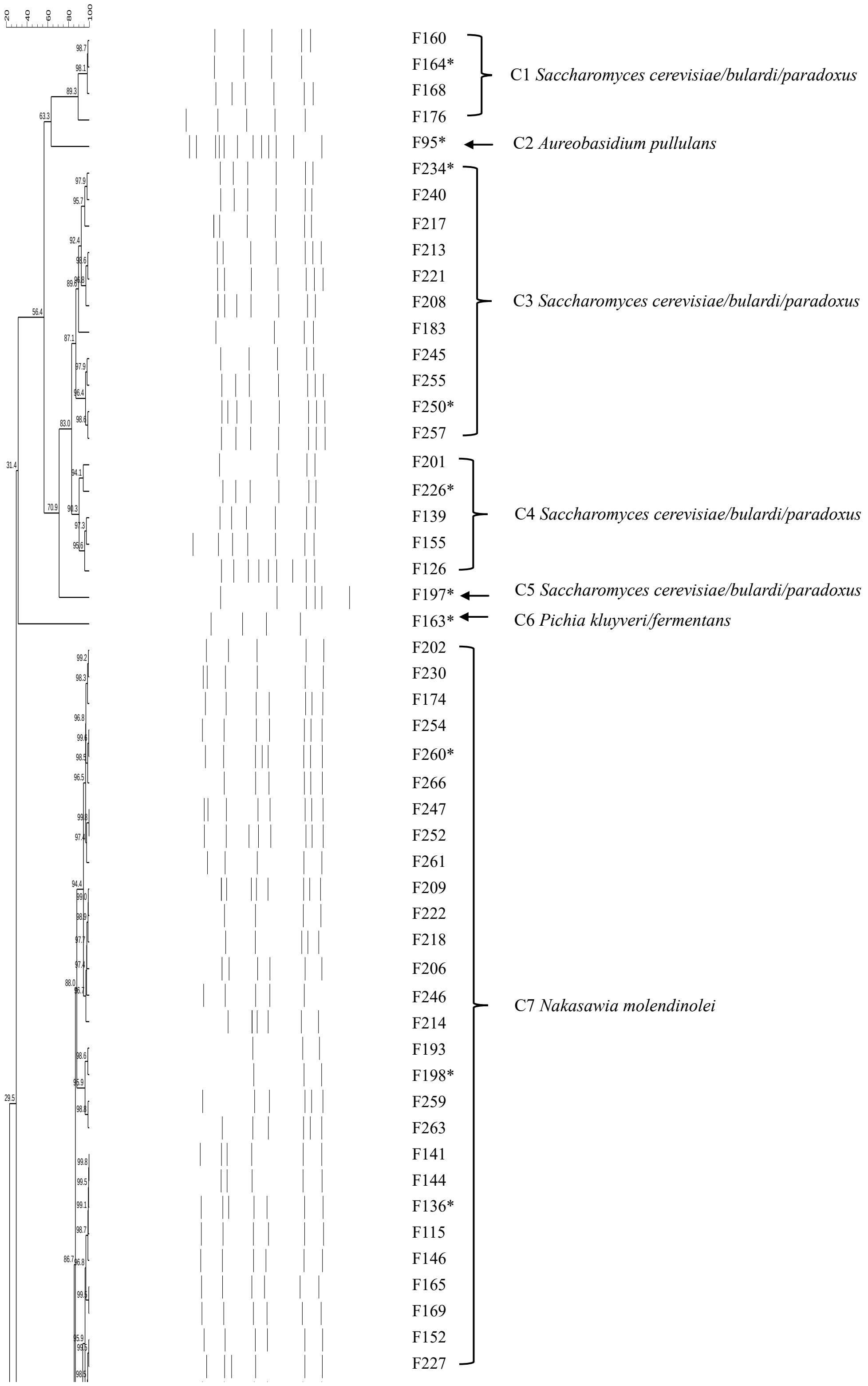
F160
F164*
F168
F176
C1 Saccharomyces cerevisiae/bulardi/paradoxus
F95*
C2 Aureobasidium pullulans
F234*
F240
F217
F213
F221
F208
F183
F245
F255
F250*
F257
C3 Saccharomyces cerevisiae/bulardi/paradoxus
F201
F226*
F139
F155
F126
C4 Saccharomyces cerevisiae/bulardi/paradoxus
F197*
C5 Saccharomyces cerevisiae/bulardi/paradoxus
F163*
C6 Pichia kluyveri/fermentans
F202
F230
F174
F254
F260*
F266
F247
F252
F261
F209
F222
F218
F206
F246
F214
F193
F198*
F259
F263
F141
F144
F136*
F115
F146
F165
F169
F152
F227
C7 Nakasawia molendinolei

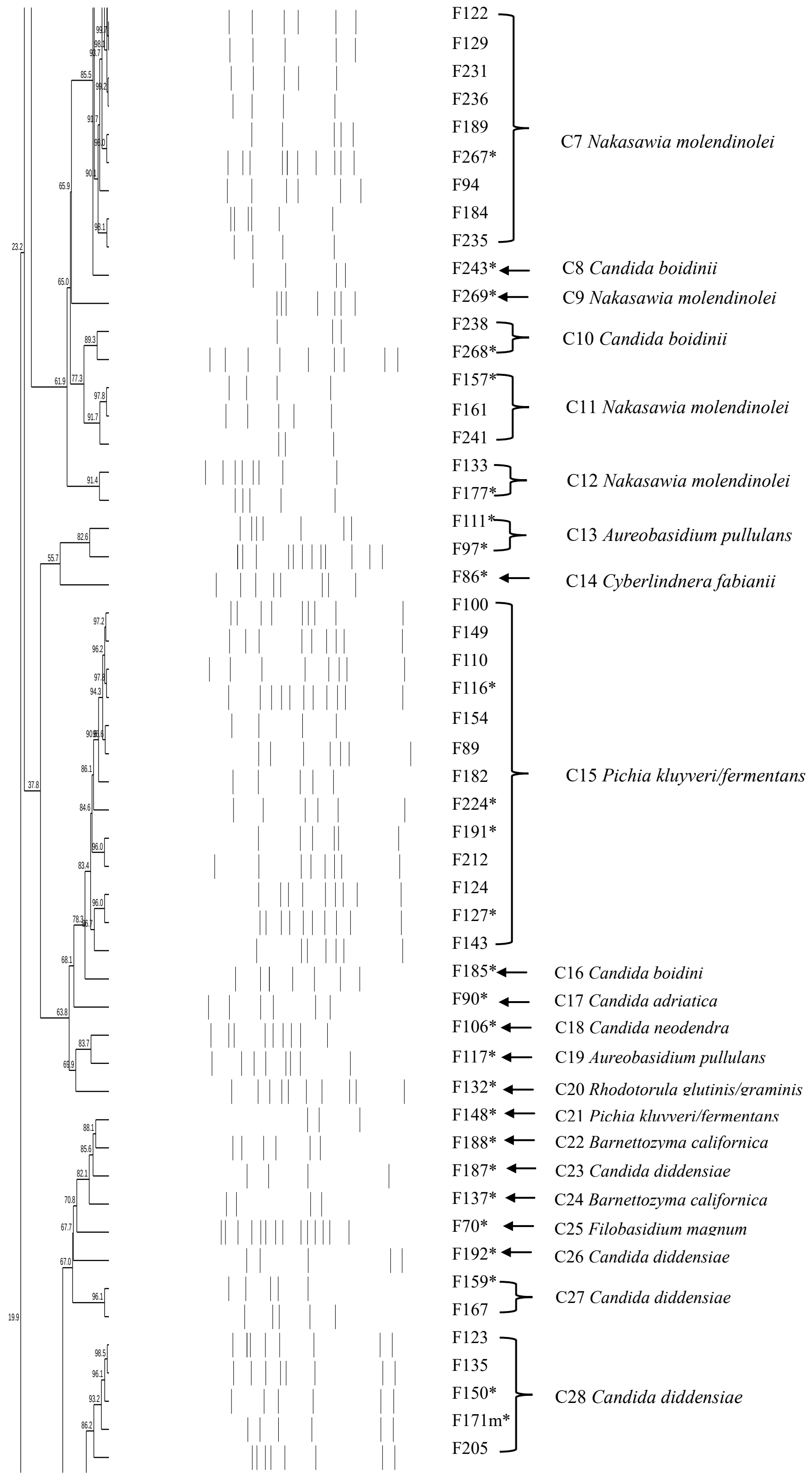

F122
F129
F231
F236
F189
F267*
F94
F184
F235
C7 Nakasawia molendinolei
F243*
C8 Candida boidinii
F269*
C9 Nakasawia molendinolei
F238
F268*
C10 Candida boidinii
F157*
F161
F241
C11 Nakasawia molendinolei
F133
F177*
C12 Nakasawia molendinolei
F111*
F97*
C13 Aureobasidium pullulans
F86*
C14 Cyberlindnera fabianii
F100
F149
F110
F116*
F154
F89
F182
F224*
F191*
F212
F124
F127*
F143
C15 Pichia kluyveri/fermentans
F185*
C16 Candida boidini
F90*
C17 Candida adriatica
F106*
C18 Candida neodendra
F117*
C19 Aureobasidium pullulans
F132*
C20 Rhodotorula glutinis/graminis
F148*
C21 Pichia kluyveri/fermentans
F188*
C22 Barnettozyma californica
F187*
C23 Candida diddensiae
F137*
C24 Barnettozyma californica
F70*
C25 Filobasidium magnum
F192*
C26 Candida diddensiae
F159*
F167
C27 Candida diddensiae
F123
F135
F150*
F171m*
F205
C28 Candida diddensiae

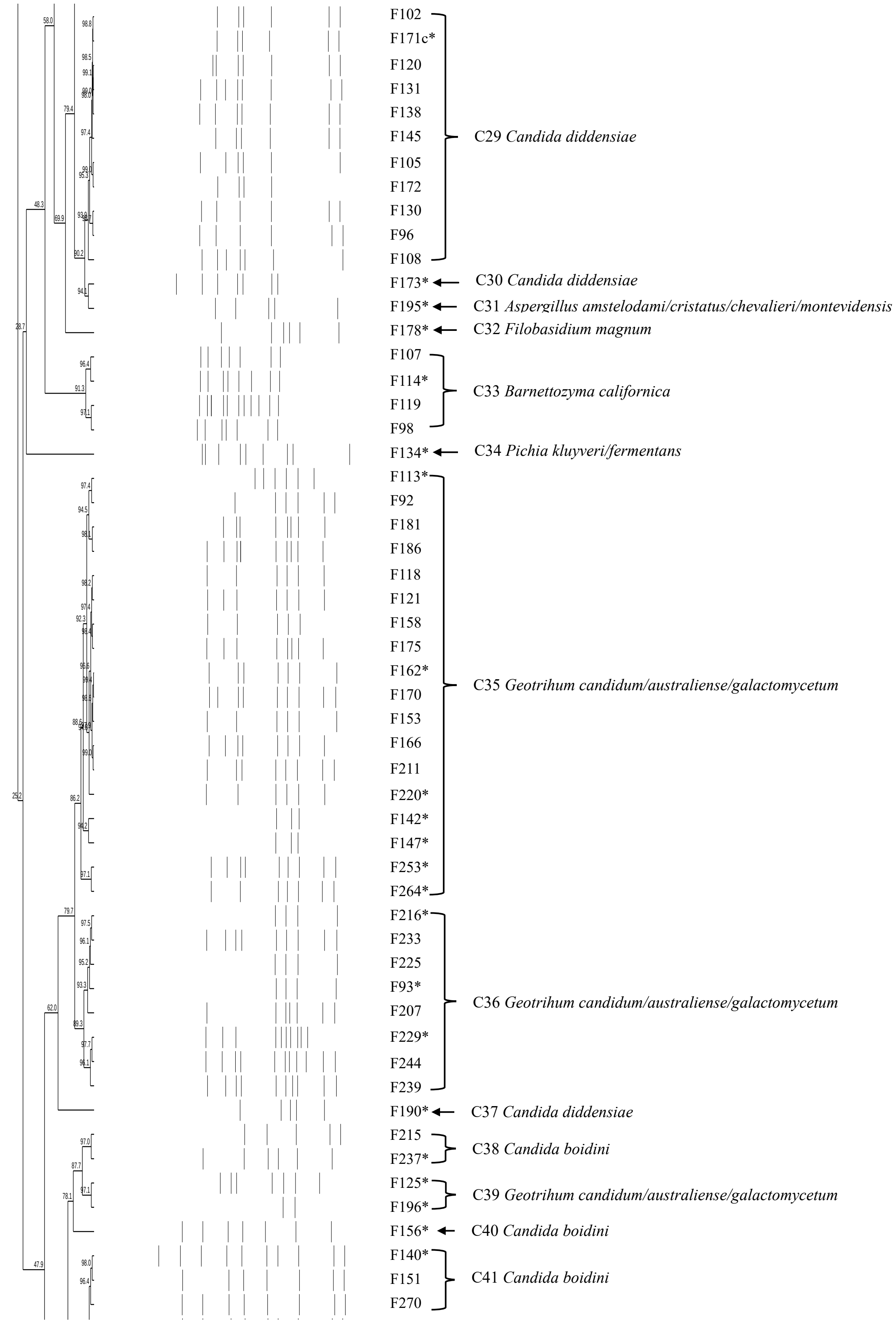
F102
F171c*
F120
F131
F138
F145
F105
F172
F130
F96
F108
C29 *Candida diddensiae*
F173*
C30 *Candida diddensiae*
F195*
C31 *Aspergillus amstelodami/cristatus/chevalieri/montevidensis*
F178*
C32 *Filobasidium magnum*
F107
F114*
F119
F98
C33 *Barnettozyma californica*
F134*
C34 *Pichia kluyveri/fermentans*
F113*
F92
F181
F186
F118
F121
F158
F175
F162*
F170
F153
F166
F211
F220*
F142*
F147*
F253*
F264*
C35 *Geotrihum candidum/australiense/galactomycetum*
F216*
F233
F225
F93*
F207
F229*
F244
F239
C36 *Geotrihum candidum/australiense/galactomycetum*
F190*
C37 *Candida diddensiae*
F215
F237*
C38 *Candida boidini*
F125*
F196*
C39 *Geotrihum candidum/australiense/galactomycetum*
F156*
C40 *Candida boidini*
F140*
F151
F270
C41 *Candida boidini*

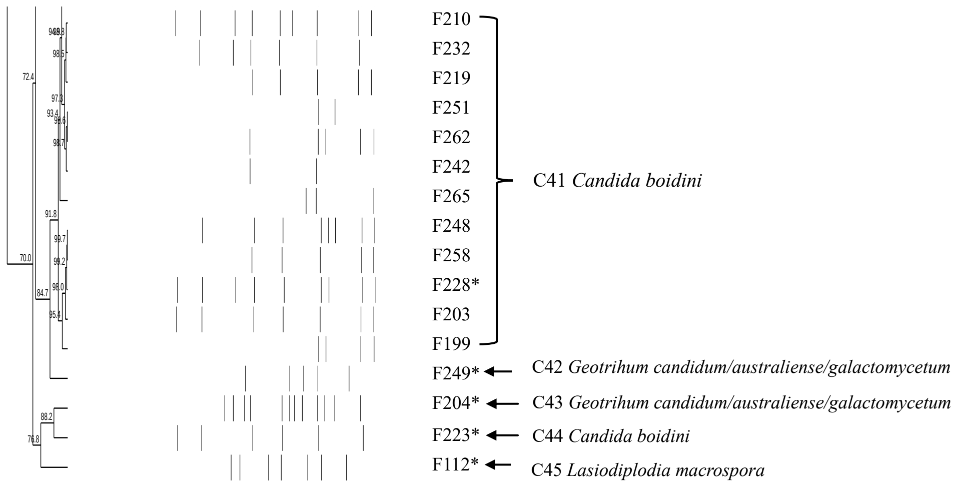
72.4
70.0
91.8
84.7
88.2
76.8
F210
F232
F219
F251
F262
F242
F265
F248
F258
F228*
F203
F199
F249*
F204*
F223*
F112*
C41 *Candida boidini*
C42 *Geotrihum candidum/australiense/galactomycetum*
C43 *Geotrihum candidum/australiense/galactomycetum*
C44 *Candida boidini*
C45 *Lasiodiplodia macrospora*
