# Supplementary figures and images for: The Natural Fermentation of Greek Tsounati Olives: Microbiome Analysis
Source: Foods. 2025 Jul 22;14(15):2568. doi: 10.3390/foods14152568 (PMC12345721; doi:10.3390/foods14152568)

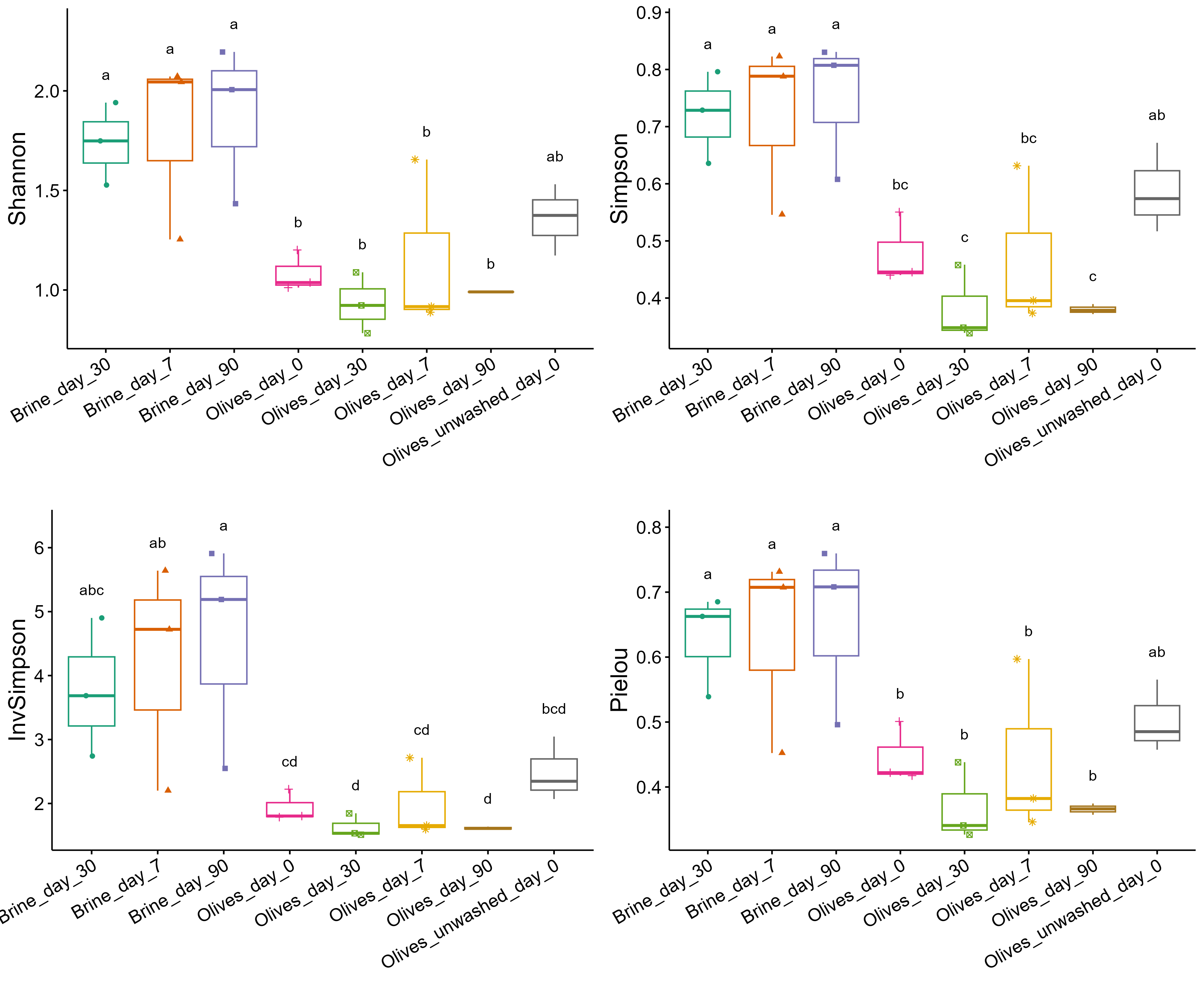

Supplement: Supplementary file 1 [file foods-14-02568-s001.zip › Figure S2.tiff]

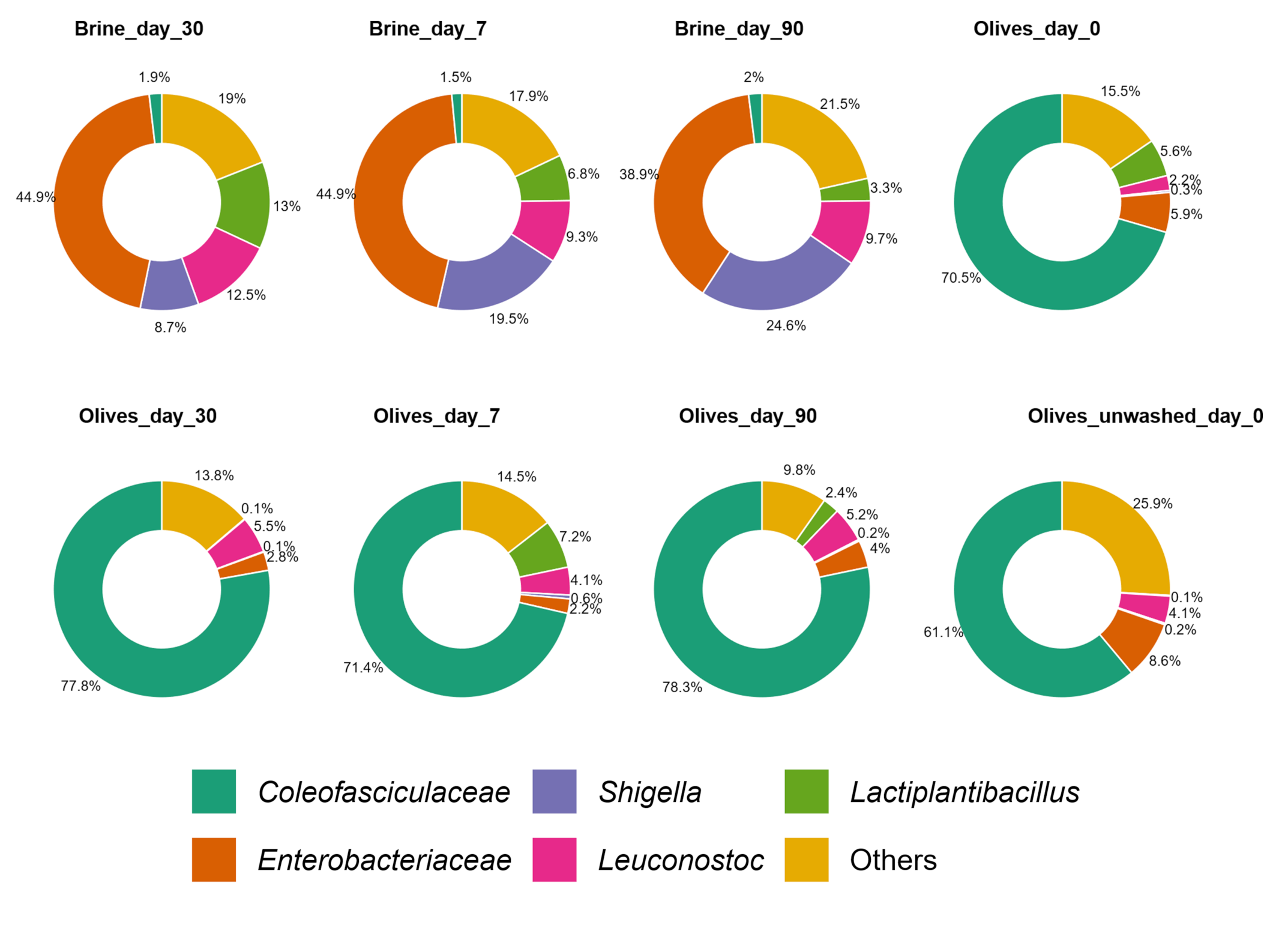

Supplement: Supplementary file 1 [file foods-14-02568-s001.zip › Figure S3.tif]

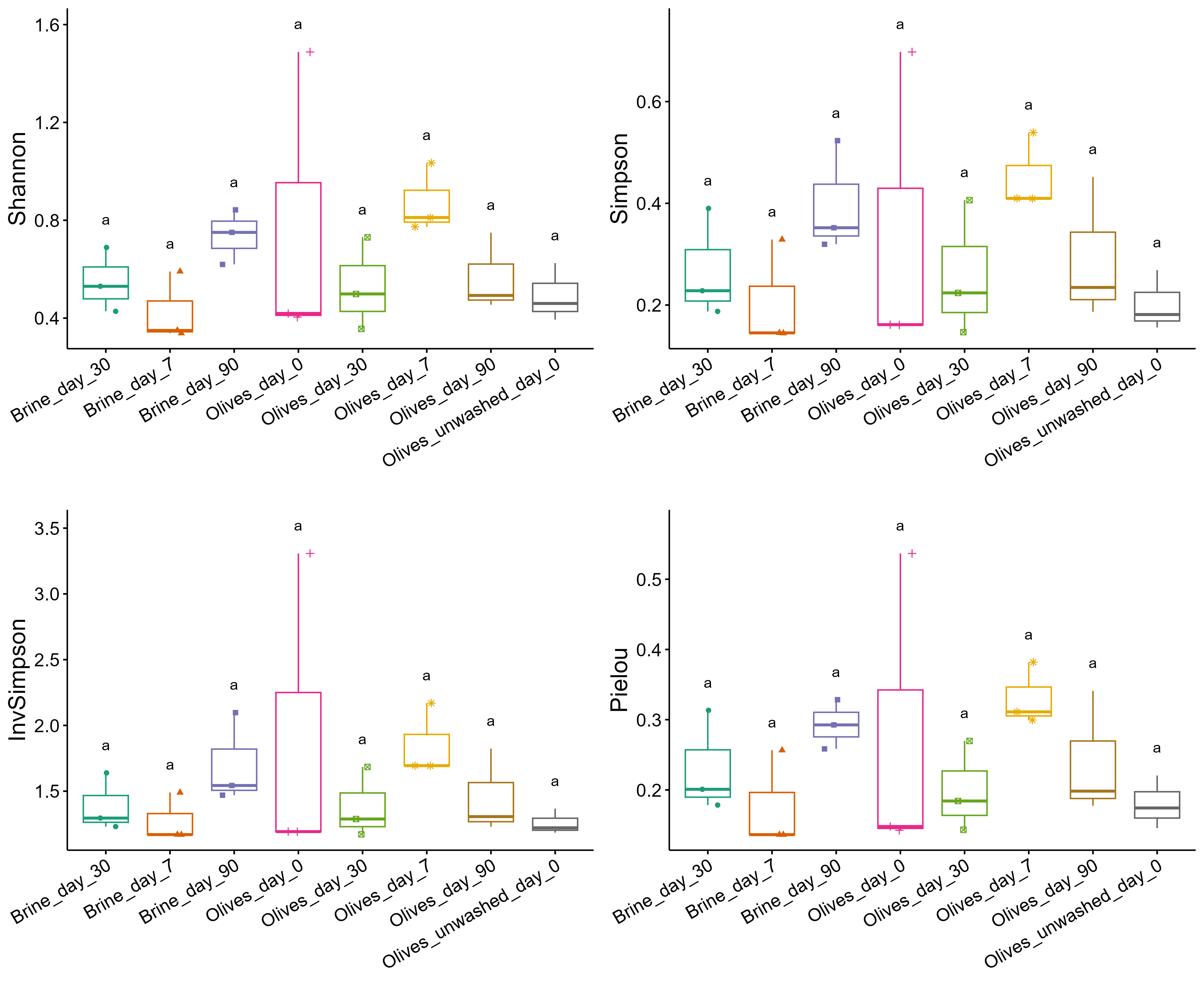

Supplement: Supplementary file 1 [file foods-14-02568-s001.zip › Figure S4.tiff]

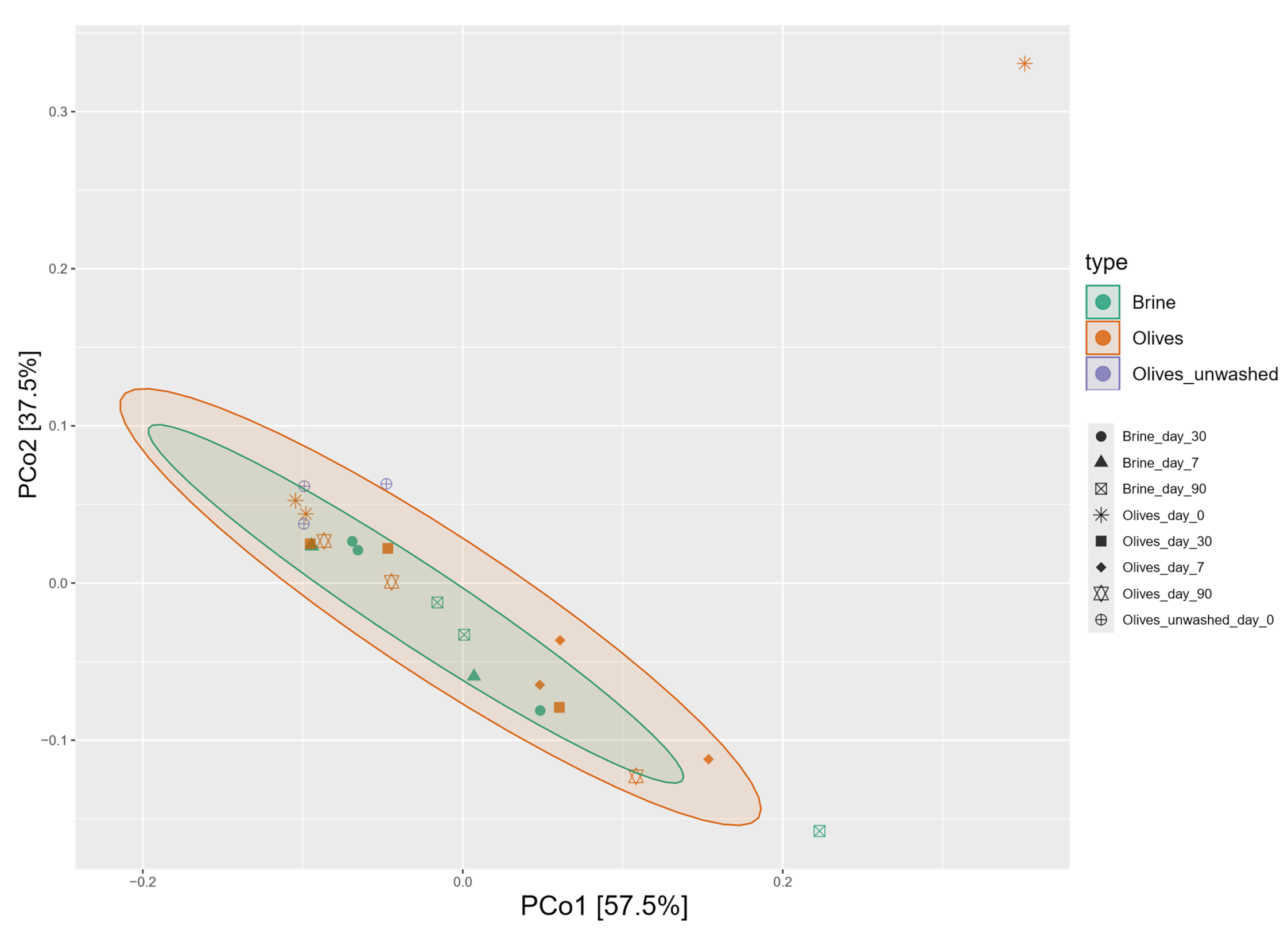

Supplement: Supplementary file 1 [file foods-14-02568-s001.zip › Figure S5.tif]
